# The Involvement of the PI3K/AKT Pathway in Zn Alleviation of Heat Stress-Induced Damage to Broiler Jejunal Organoids

**DOI:** 10.3390/ani16101492

**Published:** 2026-05-13

**Authors:** Weizhen Song, Weiyun Zhang, Xi Lin, Hsiao-Ching Liu, Jack Odle, Miles Todd See, Shengchen Wang, Xiaoyan Cui, Chuanlong Wang, Liyang Zhang, Xugang Luo

**Affiliations:** 1 Poultry Mineral Nutrition Laboratory, College of Animal Science and Technology, Yangzhou University, Yangzhou 225000, China; weizhen9697@163.com (W.S.); 15516412374@163.com (W.Z.); shengchenwang@yzu.edu.cn (S.W.); xycui@yzu.edu.cn (X.C.); wangchuanlong1994@163.com (C.W.); 2 Department of Animal Science, North Carolina State University, Raleigh, NC 27695, USA; xilin@ncsu.edu (X.L.); hc_liu@ncsu.edu (H.-C.L.); jodle@ncsu.edu (J.O.); todd_see@ncsu.edu (M.T.S.); 3 Mineral Nutrition Research Division, State Key Laboratory of Animal Nutrition, Institute of Animal Science, Chinese Academy of Agricultural Sciences, Beijing 100193, China; zhangliyang@caas.cn

**Keywords:** PI3K/AKT signaling pathway, zinc, heat stress-induced damage, broiler jejunal organoids, cell proliferation

## Abstract

The direct involvement of the phosphatidylinositol 3-kinase (PI3K)/serine threonine kinase (AKT) signaling pathway in the alleviation of the heat stress (HS)-induced damage to the integrity and barrier function of broiler jejunal organoids (JOs) by supplemental zinc (Zn) has not been confirmed. To verify it, two experiments were conducted in the present study. In experiment 1, the optimal concentrations of PI3K/AKT inhibitor (PI3K-IN-1) or agonist (YS-49) were screened. In experiment 2, the role of PI3K/AKT in Zn alleviation of HS-induced damage to JOs was evaluated with three JO types as control groups under baseline incubation temperature (40 °C) plus a 3 (JOs types) × 3 (Zn sources) factorial design under high temperature (44 °C). The results showed that the optimal concentrations of the PI3K-IN-1 and YS-49 for effectively inhibiting and promoting (*p* < 0.001) phosphorylation of PI3K and AKT were 16 μmol/L and 9 μmol/L, respectively. Adding Zn, especially Zn proteinate with moderate chelation strength (Zn-Prot M), alleviated (*p* < 0.001) the HS-induced increases in diamine oxidase content and lactate dehydrogenase activity in the media and the HS-induced decreases in JOs budding percentage, proportions of 5-ethynyl-2′-deoxyuridine and proliferating cell nuclear antigen positive cells, and the phosphorylation of PI3K and AKT. PI3K/AKT inhibition or activation reduced or enhanced (*p* < 0.05) the above alleviating effect of Zn, especially Zn-Prot M. These results indicate that the PI3K/AKT signaling pathway mediated the alleviation of HS-induced damage to integrity and barrier function of broiler JOs by supplemental Zn, particularly Zn-Prot M via promotion of cell proliferation.

## Simple Summary

The direct relationship between the phosphatidylinositol 3-kinase (PI3K)/serine threonine kinase (AKT) signaling pathway and zinc (Zn) in alleviating heat stress (HS)-induced damage to broiler intestinal organoids remains unclear. This study investigated whether Zn could protect broiler jejunal organoids (JOs) from HS-induced damage to integrity and barrier function by regulating the PI3K/AKT pathway and enhancing cell proliferation. To establish an effective mediation mechanism, the optimal concentrations of a PI3K/AKT inhibitor and an agonist for JOs were first determined. Supplemental Zn significantly alleviated the HS-induced increases in diamine oxidase content and lactate dehydrogenase activity in the media and the HS-induced decreases in budding percentage, cell proliferation, and the phosphorylation of PI3K and AKT in JOs. Inhibiting the PI3K/AKT pathway strongly reduced the protective effect of Zn, whereas activating this pathway markedly strengthened it. These results demonstrate that the PI3K/AKT signaling pathway mediates Zn alleviation of HS-induced damage to the integrity and barrier function of broiler JOs via promotion of cell proliferation.

## 1. Introduction

Heat stress (HS) has become a critical environmental constraint to broiler production efficiency, especially in intensive production [1,2]. Broilers are particularly sensitive to high temperature (HT) due to their rapid growth, high metabolism, and lack of sweat glands [3]. The gastrointestinal tract is recognized as a key organ affected by HS, particularly the jejunum in broilers [4,5]. Continuous HS can damage the intestinal integrity, reduce the efficiency of nutrient absorption, and weaken the intestinal defense against pathogenic microorganisms. These eventually impair growth performance and cause huge economic losses to the broiler industry [6,7]. Therefore, exploring effective strategies to mitigate the negative effects of HS is critical for ensuring the longevity of the broiler industry. Nutritional regulation is one of the most cost-effective approaches against HS. Among nutritional intervention strategies, the trace element zinc (Zn) has been reported to alleviate the HS-induced negative effects in broilers and other agricultural animals [8,9,10]. However, the underlying molecular mechanisms remain unclear.

To explore the above molecular mechanisms, the advanced three-dimensional (3D) intestinal organoid (IO) model should be used, because it can effectively overcome the inherent variability and uncontrollability of in vivo studies and is superior to in vitro two-dimensional (2D) intestinal epithelial cell monolayer models [11,12]. In previous work, we established a culture method for broiler jejunal organoids (JOs) and an HS-induced damage model of JOs [13,14]. Using this model, the physiological structure of the jejunum was successfully simulated in vitro, and the possible molecular mechanism by which Zn alleviated HS-induced damage to the integrity and barrier function of the jejunum in broilers was explored [13].

The intestinal physical barrier serves as the core and fundamental line of defense, and its structural integrity is a prerequisite for normal function [15]. A previous study has shown that fluorescein isothiocyanate–dextran (FITC-D) can be used as an indicator of intestinal paracellular permeability in poultry research [16]. Water supplementation with amino acid-chelated trace minerals reduced serum FITC-D levels, indicating an effective alleviation of HS-induced damage to the intestinal integrity and barrier function of broilers [17]. Additionally, the diamine oxidase (DAO) content and lactate dehydrogenase (LDH) activity are also important indicators reflecting the intestinal barrier function. After exposure to HT, the DAO content in mouse serum and LDH activity in culture medium were increased [18,19].

The stable proliferation of intestinal epithelial cells is the foundation of intestinal mucosal integrity, ensuring intestinal digestion, absorption and barrier function [20]. Proliferating cell nuclear antigen (PCNA) is closely associated with cell proliferation, and the proportion of PCNA positive cells can be used as an indicator of the cell proliferation capacity [21]. 5-ethynyl-2′-deoxyuridine (EdU) can be specifically incorporated into synthesized DNA strands and then conjugated with fluorescent azides to emit fluorescent signals, enabling the direct visualization of cell proliferation [22]. In the intestinal epithelial cell (IEC)-6 cell model, HS induces G1 phase cell cycle arrest, significantly reducing the proportion of EdU positive cells [23].

Studies have demonstrated that HS can affect the phosphatidylinositol 3-kinase (PI3K)/serine threonine kinase (AKT) signaling pathway, which plays a crucial role in the growth and self-renewal of intestinal epithelial cells [24,25]. Activation of the PI3K/AKT/mechanistic target of rapamycin (mTOR) pathway was shown to promote cell proliferation and/or improve intestinal barrier function in different cells or rats [26,27,28]. It is reported that supplemental Zn, especially Zn proteinate with moderate chelation strength (Zn-Prot M), promoted the cell proliferation and improved the barrier function in broiler primary jejunal epithelial cells (BPJECs) via this signaling pathway [29].

In our previous study, it was found that the addition of Zn, particularly Zn-Prot M, effectively relieved HS-induced damage to the integrity and barrier function of broiler JOs, possibly by promoting cell proliferation via activation of the G protein-coupled receptor 39 (GPR39)/phospholipase C beta 1 (PLCβ1)-mediated PI3K/AKT or mitogen activated protein kinase (MAPK) signaling pathways [13]. However, no study has confirmed a direct relationship between the Zn-mediated alleviation of HS-induced damage to broiler JOs and the PI3K/AKT signaling pathway. Here, we hypothesized that the PI3K/AKT signaling pathway would mediate the Zn alleviation of HS-induced damage to broiler JOs via the promotion of cell proliferation. To test this, we evaluated the effects of different Zn sources on the integrity, barrier function, cell proliferation and the phosphorylation of PI3K/AKT signaling pathway in HS-exposed broiler JOs through PI3K/AKT inhibition or activation.

## 2. Materials and Methods

### 2.1. Animal Ethics

All experiments followed the guidelines of the Animal Ethics Committee of Yangzhou University [SYXK (Su) 2021-0027].

### 2.2. Animals

A total of 20 one-day-old Arbor Acres (AA) broilers (Jiangsu Jinghai Poultry Industry Co., Ltd., Nantong, China) were maintained in one brooding chick cage with enough space and comfortable temperature and provided with ad libitum water but no feed access until the jejunal collection at 4 d of age. The sufficient crypts were isolated and pooled from 8/12 broilers for experiments 1/2.

### 2.3. Experimental Design and Treatments

Two experiments with a completely randomized design were conducted using the cultured JOs in this study.

In experiment 1, the optimal concentrations of the PI3K/AKT inhibitor (PI3K-IN-1) or agonist (YS-49) in inhibiting or promoting the phosphorylated protein expression levels of PI3K and AKT in broiler JOs were screened under the baseline incubation temperature (BIT, 40 °C) [14]. Following our previous studies [30,31,32], the concentrations of PI3K-IN-1 were set at 0, 8, 16, 24, 32 and 40 μmol/L, and the concentrations of YS-49 were set at 0, 3, 6, 9, 12 and 15 μmol/L. Thus, there were 6 treatments for either PI3K-IN-1 or YS-49, with 4 replicates for each treatment and 3 wells (1 well for JO budding percentage observation, DAO content and LDH activity assays, as well as the FITC-D permeability test, and another 2 wells for western blotting assay) for each replicate.

In experiment 2, the role of the PI3K/AKT signaling pathway in alleviating HS-induced damage to broiler JOs by Zn was investigated through PI3K/AKT inhibition or activation. Based on our previous research [30], a 3 (JO types) × 3 (Zn sources) factorial arrangement under HT (44 °C) was designed, meanwhile, 3 JO types under BIT (40 °C) were included as controls. The 3 JO types were normal (NE), PI3K/AKT-inhibited (IE) and -activated (AE) broiler JOs, and the optimal added concentrations of the PI3K/AKT inhibitor (PI3K-IN-1) or agonist (YS-49) were selected based on the results of experiment 1. The three Zn sources were no Zn addition, Zn sulfate (reagent grade, ZnSO_4_·7H_2_O, ZnSO_4_) and Zn-Prot M (feed grade, quotient of formation (Q_f_) value of 51.6, containing 17.09% of Zn by analysis), respectively. According to the categorization of Holwerda et al. [33], the Q_f_ values between 10 and 100 are defined as the moderate chelation strengths. These Zn sources were the same as in our previous studies [4,13,29,34]. The selected supplemental Zn level was 50 μmol/L, and the incubation time was 12 h based on our previous study [13]. Thus, there were 12 treatments with 6 replicates for each treatment and 7 wells (1 well for JO budding percentage observation, DAO content and LDH activity assays, as well as the FITC-D permeability test, 1 well for the EdU assay, 1 well for the PCNA assay, and another 4 wells for the Western blot assay) per replicate.

### 2.4. Preparations of Basal Media and Supplemental Zn Media for Broiler JOs

The basal media included a premixed solution, jejunal organoid growth medium (JOGM) and jejunal organoid differentiation medium (JODM).

The premixed solution contained 28.17% (*v*/*v*) penicillin/streptomycin (P/S, Gibco, Waltham, MA, USA), 28.17% (*v*/*v*) Glutamax (200 mM, Gibco), 28.17% (*v*/*v*) HEPES (1M, Gibco), 5.63% (*v*/*v*) N-Acetyl-L-Cysteine (500 mM, NAC, Sigma, Kaw, Japan), 2.82% (*v*/*v*) Y-27632 2HCl (10 mM, R&D System, Minneapolis, MN, USA), 2.82% (*v*/*v*) SB202190 (10 mM, R&D System), 2.82% (*v*/*v*) A83-01 (0.5 mM, Sigma), and 1.40% (*v*/*v*) EGF (100 μg/mL, Gibco).

JOGM contained 50% (*v*/*v*) L-WRN conditioned medium, 36.45% (*v*/*v*) Advanced DMEM/F12 (Gibco), 10% (*v*/*v*) fetal bovine serum (FBS, Gibco), and 3.55% (*v*/*v*) premixed solution. The L-WRN conditioned medium was prepared as described previously [14].

JODM contained 96.45% (*v*/*v*) Advanced DMEM/F12, 3.55% (*v*/*v*) premixed solution, 50 ng/mL RSPO1 (XianJue Biotechnology, Suzhou, China), 50 ng/mL Noggin (XianJue Biotechnology), and 2.5 μM CHIR99021 (MCE, Junction, NJ, USA).

The supplemental Zn media were prepared by adding either ZnSO_4_ or Zn-Prot M to the above JODM at 50 μmol/L of supplemental Zn level. The analyzed Zn concentrations in the media for all of treatment groups are listed in Table 1.

### 2.5. Isolation of Jejunal Crypts and Cultivation of Broiler JOs

The jejunal crypts were isolated following our previous protocol [13,14]. Briefly, the jejunum was harvested from 20 (8 in experiment 1 and 12 in experiment 2) 4-day-old broilers. Then the jejunum was rinsed, cut into fragments, and digested. The crypts were collected through filtering and centrifuging. Finally, the collected crypts were resuspended in growth factor-reduced Matrigel (BD, Franklin Lakes, NJ, USA), pipetted into 24-well plates, and incubated under BIT for 10 min. After 10 min incubation, JOGM was added to each well, and the JOs were cultivated under BIT for 3 d.

### 2.6. JOs Treatments and Cultivation

In experiment 1, the JOGM was replaced with 500 μL of fresh JODM after 3 d, the JOs were cultivated continuously under BIT for 36 h, and then, the PI3K-IN-1 or YS-49 was added into different wells according to the experimental design and treatments. The JOs then were cultivated under the same conditions for another 12 h.

**Figure 1:**
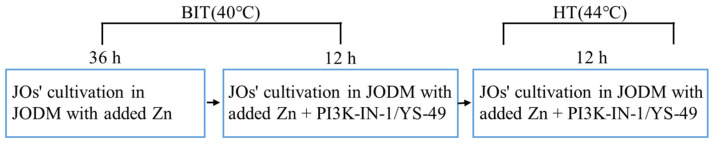
The diagram of the protocol in experiment 2. BIT: baseline incubation temperature. HT: high temperature. JOs: jejunal organoids. JODM: jejunal organoid differentiation medium. PI3K-IN-1: PI3K/AKT inhibitor. YS-49: PI3K/AKT agonist.

In experiment 2, the JOGM was replaced with 500 μL of fresh JODM containing added Zn as either ZnSO_4_ or Zn-Prot M after 3 d. Similar to experiment 1, the JOs were cultivated under BIT for 36 h, and then PI3K-IN-1 or YS-49 was added to the different wells to continuously cultivate the JOs for another 12 h. At the end of the 12 h cultivation, the JOs for the HT treatment groups were cultivated at 44 °C for 12 h, while those for the BIT groups continued to be cultivated at 40 °C for 12 h. The diagram of this protocol is presented in Figure 1.

### 2.7. Calculation of JOs’ Budding Percentage

At the end of the JO treatments and cultivation, the numbers of total JOs and JOs with budding structures in 1 well from each replicate of each treatment in two experiments were counted under an inverted microscope, and the budding percentage was calculated as the number of JOs with budding structure/the number of total JOs × 100% [13,14].

### 2.8. Sample Collections and Preparations

At the end of JO treatments and cultivation, as described previously [13], the medium was collected and centrifuged. The supernatants were then transferred to new tubes and stored at −20 °C for analyses of the DAO content and LDH activity.

After removing the media in all wells, the protocols for Matrigel digestion and JO isolation were the same as in previous studies [13,14]. The harvested JOs were used for determining the FITC-D permeability, cell proliferation, and phosphorylation of PI3K and AKT.

### 2.9. FITC-D Permeability

The FITC-D permeability was measured following the procedure described previously [13,14]. Briefly, the harvested JOs from 1 well in each replicate of each treatment in two experiments were incubated in 25 μg/L of FITC-D (40 kDa, Sigma-Aldrich, Burlington, MA, USA) diluted in phenol red free DMEM at 25 °C for 1 h and then observed under the fluorescence inverted microscope (Olympus, Hachioji, Japan) to capture the images.

### 2.10. DAO Content and LDH Activity in Media

According to the manufacturers’ instructions, the DAO content (Jianglai Biotechnology, Shanghai, China) and the LDH activity (Nanjing Jiancheng Bioengineering Institute, Nanjing, China) in the media were measured, respectively.

### 2.11. Cell Proliferation

The cell proliferation was determined as described previously [13,35,36]. Briefly, the collected JOs from experiment 2 were subjected to EdU staining, according to the Cell-Light EdU Apollo567 In Vitro Kit (RIBOBIO, Guangzhou, China) instructions. The immunofluorescence (IF) staining protocols of PCNA in the collected JOs from experiment 2 were as follows: JOs were sequentially fixed, permeabilized, and blocked. Samples were then incubated with PCNA primary antibodies (ABclonal, Beijing, China) and Alexa Fluor 594-conjugated secondary antibodies (ABclonal), followed by DAPI nuclear staining. Fluorescence images were acquired via a laser-scanning confocal microscope (Olympus). For quantitative analysis, the mean densities of EdU-positive, PCNA-positive, and total cells were determined using ImageJ (v1.50) software. Finally, the proportions of EdU- and PCNA-positive cells in the total cells were calculated as the ratio of the red fluorescence density of EdU-positive or PCNA-positive cells to the blue fluorescence density of the total cells times 100% to evaluate cell proliferation.

### 2.12. Western Blot

The Western blot assay was performed following the protocols as described previously [13,14]. Briefly, total and phosphorylated proteins were extracted from harvested JOs using ice-cold RIPA lysis buffer supplemented with a protease and phosphatase inhibitor cocktail. The concentrations of total and phosphorylated proteins were determined and adjusted by the BCA method (Thermo Fisher, Waltham, MA, USA). Equal amounts of protein per lane were separated by 10% sodium dodecyl sulfate–polyacrylamide gel electrophoresis (SDS-PAGE) and transferred by electrotransfer onto nitrocellulose membranes (Merck Millipore, Burlington, MA, USA). Membranes were then blocked with 5% (*w*/*v*) bovine serum albumin (BSA) in Tris-buffered saline with Tween 20 (TBST) to block non-specific binding. Subsequently, membranes were incubated with primary antibodies (antibody details are listed in Table 2), washed with TBST, and then incubated with HRP-conjugated goat anti-rabbit secondary antibodies. Protein signals were visualized using an enhanced chemiluminescence (ECL) detection system (Tanon, Shanghai, China), and band intensities were quantified with Gis 1D software (v4.2, Tanon). The values of phosphorylated protein expression levels of target genes were calculated as the relative quantities (RQ) of phosphorylated protein band intensities of target genes to their corresponding protein band intensities.

### 2.13. Statistical Analyses

All data were statistically analyzed using SAS (2013) software (version 9.4, SAS Institute Inc., Cary, NC, USA). The data from experiment 1 and the data of the 3 JO types under BIT in experiment 2 were analyzed using one-way ANOVA via the Mixed Model procedure. Orthogonal comparisons were performed also for the data from experiment 1 to test the linear or quadratic effects of different concentrations of the inhibitor or agonist on measured variables [30]. For the data from experiment 2, T-tests were conducted to compare the significant differences between BIT and HT for each of the 3 JO types. Two-way ANOVA was applied for the data obtained under HT, with the statistical model including JO type, Zn source, and their interaction. The significant differences among means were compared using the post hoc Tukey’s HSD method [14]. Data of budding percentages and the proportions of EdU and PCNA positive cells were transformed to arcsine for analyses. The replicate well or the pooled replicate well sample was used as an experimental unit, and the statistical significance was set at *p* < 0.05.

## 3. Results

### 3.1. Effects of PI3K/AKT Inhibitor and Agonist Concentrations on the Integrity, Barrier Function and Budding Percentage of Broiler JOs (Experiment 1)

**Figure 2:**
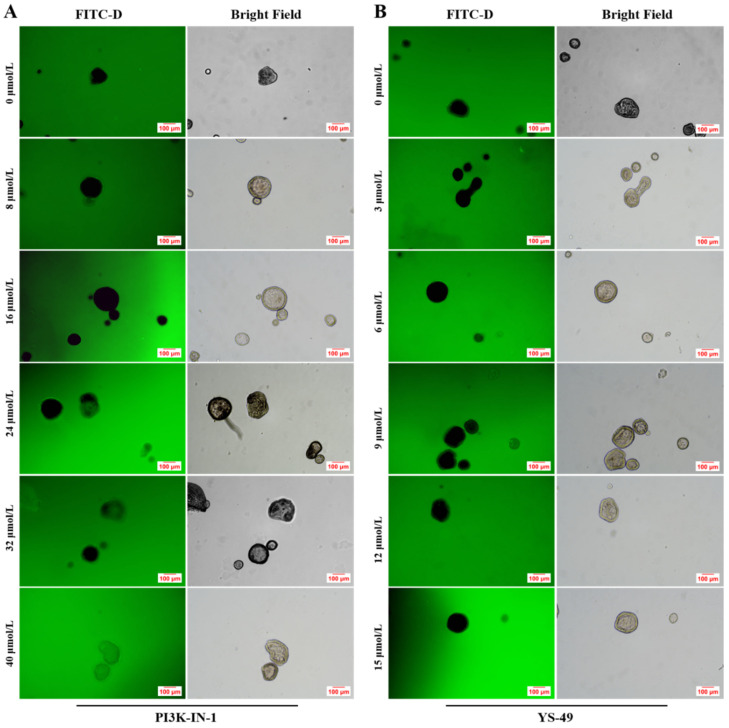
Effects of PI3K-IN-1 and YS-49 concentrations on the integrity of broiler JOs under HS (experiment 1). (**A**) Integrity of broiler JOs with PI3K-IN-1. (**B**) Integrity of broiler JOs with YS-49. PI3K-IN-1: PI3K/AKT inhibitor. YS-49: PI3K/AKT agonist. JOs: jejunal organoids. FITC-D 40: fluorescein isothiocyanate–dextran, 40 kDa. Scale bar = 100 μm.

As shown in Figure 2A, the addition of 8 and 16 μmol/L of PI3K-IN-1 had no effect on the integrity of JOs, with no green fluorescence observed in the JOs. However, when the supplemental concentrations reached 24, 32 and 40 μmol/L, green fluorescence was observed in the lumen of Jos, and the fluorescence intensity increased as the concentrations of PI3K-IN-1 increased. As shown in Figure 2B, the addition of different concentrations of YS-49 did not affect the integrity of JOs as evidenced by the lack of FITC-D permeation into JOs. As for the FITC-D test, unfortunately, we were only able to rely on qualitative visual imagery to assess the extent of the JO barrier integrity damage and unable to quantify it.

**Figure 3:**
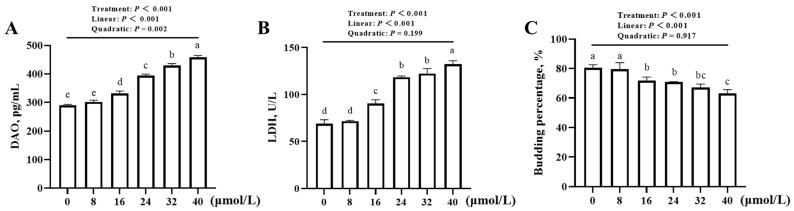
Effect of PI3K-IN-1 concentration on the barrier function and budding percentage of broiler JOs (experiment 1). (**A**) DAO content in the medium. (**B**) LDH activity in the medium. (**C**) Budding percentage of broiler JOs. JOs: jejunal organoids. DAO: diamine oxidase. LDH: lactate dehydrogenase. PI3K-IN-1: PI3K/AKT inhibitor. Different letters (a~e) indicate significant differences (*p* < 0.05) among treatments. Data are means ± SE (n = 3–4). N = 3–4, because there is one replicate outlier for some treatments, and we adopted the commonly-used interquartile range (IQR) method to eliminate outliers within each group. The same is true for that below.

The addition of different concentrations of PI3K-IN-1 exerted an effect (*p* < 0.001) on the DAO content, LDH activity in the media and the budding percentage of JOs as shown in Figure 3A–C. With the increase in the PI3K-IN-1 concentrations, the DAO contents and LDH activities increased linearly (*p* < 0.001), while the budding percentages decreased linearly (*p* < 0.001). A quadratic increase (*p* = 0.002) was also observed for the DAO contents. Compared with no addition control, the minimum supplemental level of PI3K-IN-1 for affecting (*p* < 0.05) the above indices was 16 μmol/L.

**Figure 4:**
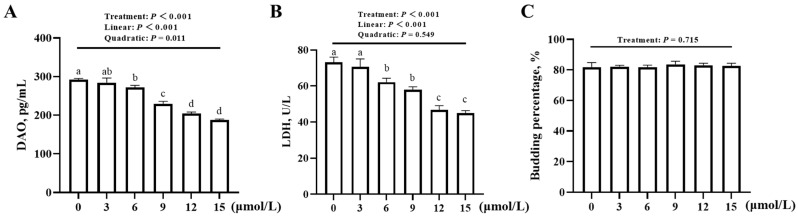
Effect of YS-49 concentration on the barrier function and budding percentage of broiler JOs (experiment 1). (**A**) DAO content in the medium. (**B**) LDH activity in the medium. (**C**) Budding percentage of broiler JOs. JOs: jejunal organoids. DAO: diamine oxidase. LDH: lactate dehydrogenase. YS-49: PI3K/AKT agonist. Different letters (a~d) indicate significant differences (*p* < 0.05) among treatments. Data are means ± SE (n = 3–4).

The addition of YS-49 had no effect (*p* = 0.715) on the budding percentage of JOs but affected (*p* < 0.001) the DAO content and LDH activity in the media (Figure 4A–C). With increasing YS-49 concentrations, the DAO contents and LDH activities decreased linearly (*p* < 0.001). A quadratic decrease (*p* = 0.011) was also detected for the DAO contents. Compared with no addition control, the minimum supplemental level of YS-49 for decreasing (*p* < 0.05) DAO content and LDH activity was 6 μmol/L.

### 3.2. Effects of PI3K/AKT Inhibitor and Agonist Concentrations on the Phosphorylated Protein Expression Levels of PI3K and AKT in Broiler JOs (Experiment 1)

**Figure 5:**
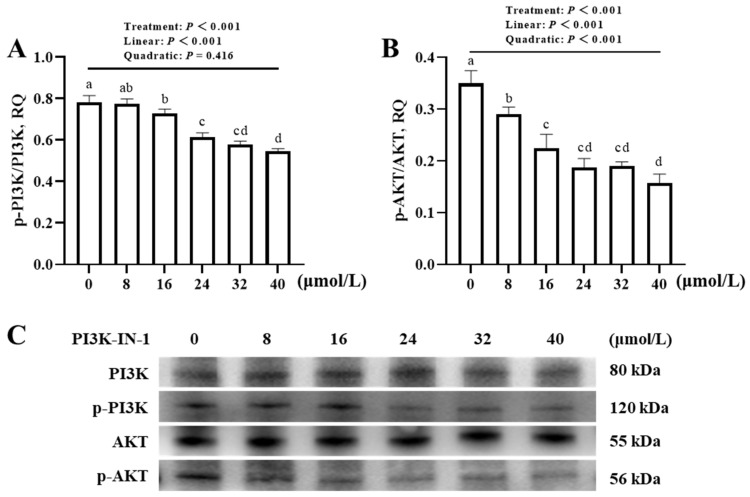
Effect of PI3K-IN-1 concentration on phosphorylated protein expression levels of PI3K and AKT of broiler JOs (experiment 1). (**A**) Phosphorylated protein expression level of PI3K. (**B**) Phosphorylated protein expression level of AKT. (**C**) Representative immunoblots. JOs: jejunal organoids. PI3K-IN-1: PI3K/AKT inhibitor. PI3K: Phosphatidylinositol 3-kinase. AKT: serine threonine kinase. Values of phosphorylated protein expression levels of target genes were calculated as the relative quantities (RQ) of phosphorylated protein band intensities of target genes to their corresponding protein band intensities. Different letters (a~d) indicate significant differences (*p* < 0.05) among treatments. Data are means ± SE (n = 3–4).

The concentration of PI3K-IN-1 affected (*p* < 0.001) the phosphorylated protein expression levels of PI3K and AKT in JOs (Figure 5A,B). With increasing PI3K-IN-1 concentrations, the phosphorylation of both PI3K and AKT decreased linearly (*p* < 0.001). A quadratic decrease (*p* < 0.001) was also detected for the phosphorylation of AKT. Compared with no addition control, the minimum supplemental levels of PI3K-IN-1 for inhibiting (*p* < 0.001) the phosphorylation of PI3K and AKT were 16 and 8 μmol/L, respectively.

**Figure 6:**
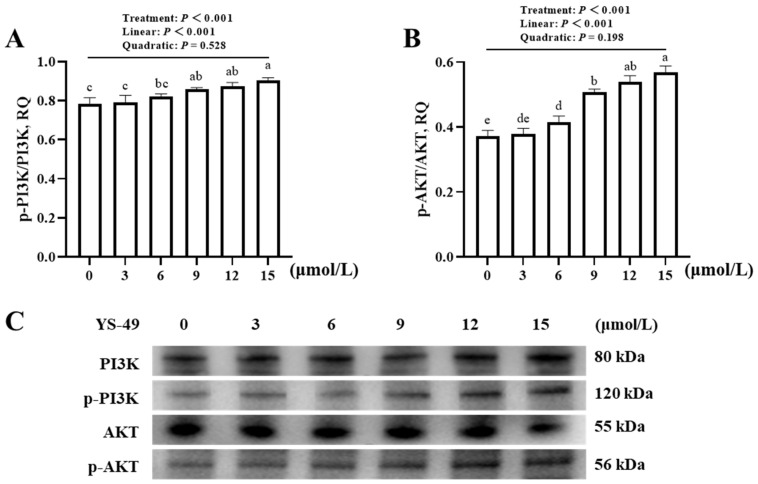
Effect of YS-49 concentration on phosphorylated protein expression levels of PI3K and AKT of broiler JOs (experiment 1). (**A**) Phosphorylated protein expression level of PI3K. (**B**) Phosphorylated protein expression level of AKT. (**C**) Representative immunoblots. JOs: jejunal organoids. YS-49: PI3K/AKT agonist. PI3K: Phosphatidylinositol 3-kinase. AKT: serine threonine kinase. Values of phosphorylated protein expression levels of target genes were calculated as the relative quantities (RQ) of phosphorylated protein band intensities of target genes to their corresponding protein band intensities. Different letters (a~e) indicate significant differences (*p* < 0.05) among treatments. Data are means ± SE (n = 3–4).

Figure 6A,B showed that the concentration of YS-49 influenced (*p* < 0.001) the phosphorylated protein expression levels of PI3K and AKT in JOs. With increasing YS-49 concentrations, the phosphorylation of both PI3K and AKT increased linearly (*p* < 0.001). Compared with no addition control, the minimum supplemental levels of YS-49 for promoting (*p* < 0.05) the phosphorylation of PI3K and AKT were 9 and 6 μmol/L, respectively.

### 3.3. Effects of Different Zn Sources on the Integrity, Barrier Function and Budding Percentage of PI3K/AKT-Normal and -Inhibited or -Activated Broiler JOs Under HS (Experiment 2)

**Figure 7:**
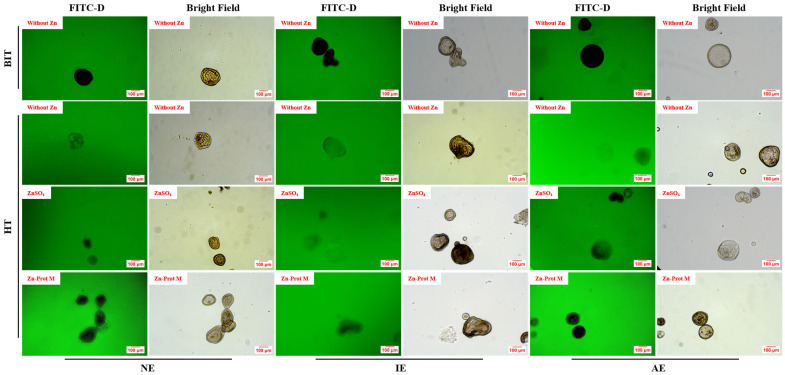
Effects of different Zn sources on the integrity of PI3K/AKT-normal and -inhibited or -activated broiler JOs under HS (experiment 2). JOs: jejunal organoids. FITC-D 40: fluorescein isothiocyanate–dextran, 40 kDa. PI3K: Phosphatidylinositol 3-kinase. AKT: serine threonine kinase. Zn-Prot M: Zn proteinate with moderate chelation strength (Q_f_ = 51.6). Supplemental Zn level: 50 μmol/L. NE: normal expression. IE: inhibited expression. AE: activated expression. Scale bar = 100 μm.

Figure 7 showed that under BIT, the JOs in the NE, IE and AE groups exhibited intact boundaries with no green fluorescence uptake. Under HT, without Zn supplementation, all three JO types showed obviously damaged boundaries and green fluorescence in the lumen. When ZnSO_4_ was added to the media under HT, the green fluorescence intensity in the lumens of NE and AE JOs was noticeably reduced compared with those without Zn addition. However, the integrity of IE JOs was similar to those without Zn addition under HT, with a minor effect on the alleviation of integrity impairment. Supplemental Zn-Prot M markedly alleviated the damage to the integrity of NE and AE JOs under HT, while the effect on the alleviation of IE JOs integrity impairment was less pronounced.

**Figure 8:**
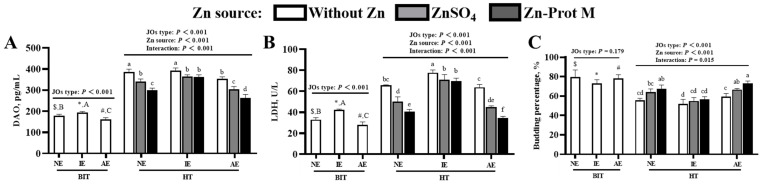
Effects of different Zn sources on the barrier function and budding percentage of PI3K/AKT-normal and -inhibited or -activated broiler JOs under HS (experiment 2). (**A**) DAO content in the medium. (**B**) LDH activity in the medium. (**C**) Budding percentage of broiler JOs. JOs: jejunal organoids. PI3K: Phosphatidylinositol 3-kinase. AKT: serine threonine kinase. BIT: baseline incubation temperature, 40 °C. HT: high temperature, 44 °C. DAO: diamine oxidase. LDH: lactate dehydrogenase. Zn-Prot M: Zn proteinate with moderate chelation strength (Q_f_ = 51.6). Supplemental Zn level: 50 μmol/L. NE: normal expression. IE: inhibited expression. AE: activated expression. Different symbols ($, *, #) indicate significant differences (*p* < 0.05) between BIT and HT for the same JO type with no Zn addition. Different letters (A, B, C) indicate significant differences (*p* < 0.05) among the three JO types under BIT. Different letters (a~f) indicate significant differences (*p* < 0.05) among treatments under HT. Data are means ± SE (n = 5–6). N = 5–6, because there is one replicate outlier for some treatments, and we adopted the commonly-used interquartile range (IQR) method to eliminate outliers within each group. The same is true for that below.

As shown in Figure 8A–C, under BIT, the JO type affected (*p* < 0.001) the DAO content and LDH activity in the media but had no effect (*p* > 0.17) on the budding percentage of JOs. Compared with the NE JOs, the IE JOs showed an increased DAO content and LDH activity (*p* < 0.001), whereas the AE JOs exhibited a decrease (*p* < 0.001) in these two indices. In comparison with the three JO types under BIT, the three JO types without Zn supplementation under HT had increased DAO contents and LDH activities (*p* < 0.001), as well as decreased budding percentages of JOs (*p* < 0.001). The JO type, Zn source and their interaction influenced (*p* < 0.02) the DAO content, LDH activity and budding percentage of JOs under HT. For NE JOs, supplemental Zn, especially Zn-Prot M, decreased (*p* < 0.05) the DAO content and LDH activity and increased (*p* < 0.05) the budding percentage of JOs compared to no Zn supplementation. For IE JOs, supplemental Zn still reduced (*p* < 0.05) the DAO content and LDH activity, with no effect (*p* > 0.05) on the budding percentage; however, the reduction resulting from supplemental Zn, especially Zn-Prot M, was lower (*p* < 0.05) than those for NE JOs; for AE JOs, added Zn, particularly Zn-Prot M, further decreased (*p* < 0.05) the DAO content and LDH activity and increased (*p* < 0.05) the budding percentage, while the decreasing or increasing effect was further expanded (*p* < 0.05) compared to NE JOs.

### 3.4. Effects of Different Zn Sources on the Cell Proliferation of PI3K/AKT-Normal and -Inhibited or -Activated Broiler JOs Under HS (Experiment 2)

**Figure 9:**
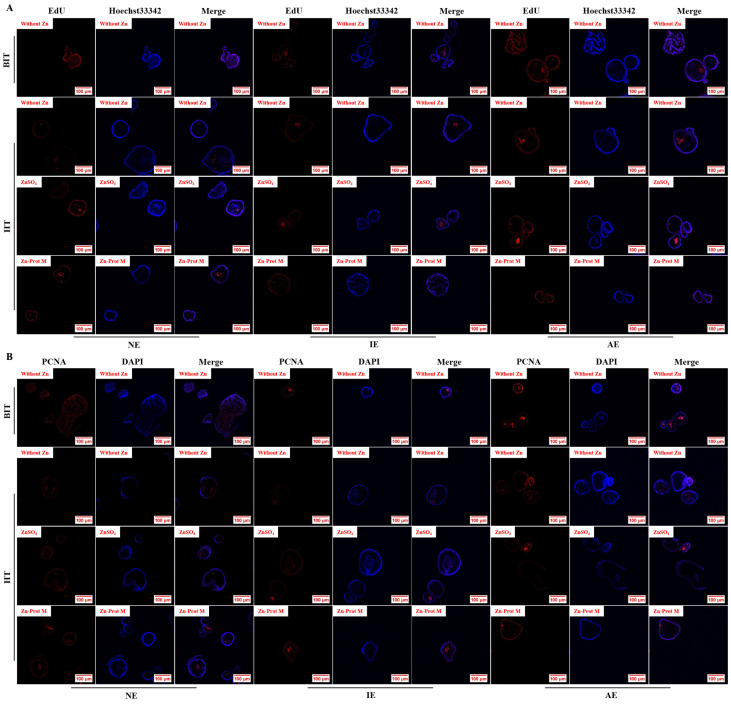
Effects of different Zn sources on the cell proliferation of PI3K/AKT-normal and -inhibited or -activated broiler JOs under HS (experiment 2). (**A**) EdU staining. (**B**) IF staining of PCNA. Nuclei in total cells were stained (blue) with Hoechst33342 (EdU) or DAPI (PCNA) to reflect the number of EdU or PCNA total cells. JOs: jejunal organoids. EdU: 5-ethynyl-2′-deoxyuridine. PCNA: proliferating cell nuclear antigen. Zn-Prot M: Zn proteinate with moderate chelation strength (Q_f_ = 51.6). Supplemental Zn level: 50 μmol/L. PI3K: Phosphatidylinositol 3-kinase. AKT: serine threonine kinase. BIT: baseline incubation temperature, 40 °C. HT: high temperature, 44 °C. NE: normal expression. Data are means ± SE (n = 6).

**Figure 10:**
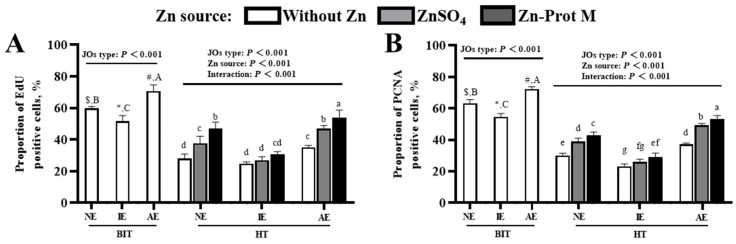
Effects of different Zn sources on the proportions of EdU and PCNA positive cells in PI3K/AKT-normal and -inhibited or -activated broiler JOs under HS (experiment 2). (**A**) Proportion of EdU positive cells. (**B**) Proportion of PCNA positive cells. JOs: jejunal organoids. EdU: 5-ethynyl-2′-deoxyuridine. PCNA: proliferating cell nuclear antigen. Zn-Prot M: Zn proteinate with moderate chelation strength (Q_f_ = 51.6). Supplemental Zn level: 50 μmol/L. PI3K: Phosphatidylinositol 3-kinase. AKT: serine threonine kinase. BIT: baseline incubation temperature, 40 °C. HT: high temperature, 44 °C. NE: normal expression. IE: inhibited expression. AE: activated expression. Different symbols ($, *, #) indicate significant differences (*p* < 0.05) between BIT and HT for the same JO type with no Zn addition. Different letters (A, B, C) indicate significant differences (*p* < 0.05) among the three JO types under BIT. Different letters (a~g) indicate significant differences (*p* < 0.05) among treatments under HT. Data are means ± SE (n = 5–6).

Under BIT, there was no obvious difference in the red fluorescence signal of EdU positive cells among the three JO types (Figure 9A). In comparison with the three JO types under BIT, the three JO types with no Zn supplementation under HT had reduced red fluorescence signal of EdU positive cells. Zn supplementation obviously enhanced the red fluorescence signal of EdU positive cells in the three JO types compared with no Zn supplementation under HT. As shown in Figure 9B, the results of the PCNA IF staining were consistent with those of EdU staining. The specific quantitative results for Figure 9 are shown in Figure 10.

Under BIT, the JO type had an effect (*p* < 0.001) on the proportion of EdU-positive cells (Figure 10A). Compared with the NE JOs, the IE JOs decreased (*p* < 0.001) it, while the AE JOs increased (*p* < 0.001) it. Under HT, the three JO types with no Zn addition exhibited a reduction (*p* < 0.001) in this index compared with those under BIT. The JO type, Zn source and their interaction had effects (*p* < 0.001) on it under HT. As for NE JOs, added Zn, especially Zn-Prot M, increased (*p* < 0.05) it compared to no Zn addition; for IE JOs, Zn supplementation had no effect (*p* > 0.05) on it; for AE JOs, supplemental Zn, particularly Zn-Prot M, further increased (*p* < 0.05) it.

The effect of the JO type on the proportion of PCNA-positive cells under BIT and the effect of HT on it in the three JO types without supplemental Zn (Figure 10B) were consistent with the above EdU staining results. The JO type, Zn source and their interaction affected (*p* < 0.001) it under HT. As for NE and AE JOs, added Zn, especially Zn-Prot M, increased (*p* < 0.05) it compared to no Zn addition, and the increasing effect was further expanded (*p* < 0.05) in AE JOs compared to NE JOs; however, for IE JOs, only Zn-Prot M increased (*p* < 0.05) it, and the increasing effect was strongly inhibited (*p* < 0.05).

### 3.5. Effects of Different Zn Sources on the Phosphorylated Protein Expression Levels of PI3K and AKT in PI3K/AKT-Normal and -Inhibited or -Activated Broiler JOs Under HS (Experiment 2)

**Figure 11:**
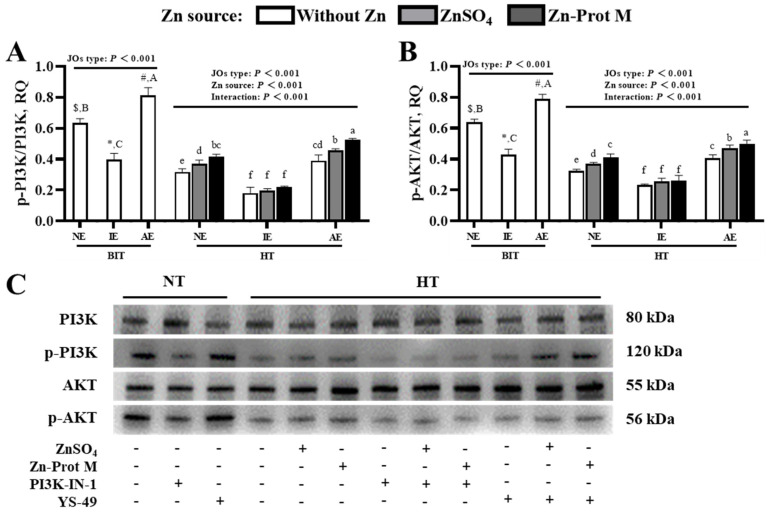
Effects of different Zn sources on phosphorylated protein expression levels of PI3K and AKT in PI3K/AKT-normal and -inhibited or -activated broiler JOs under HS (experiment 2). (**A**) Phosphorylated protein expression level of PI3K. (**B**) Phosphorylated protein expression level of AKT. (**C**) Representative immunoblots. JOs: jejunal organoids. NE: normal expression. IE: inhibited expression. AE: activated expression. Zn-Prot M: Zn proteinate with moderate chelation strength (Q_f_ = 51.6). Supplemental Zn level: 50 μmol/L. PI3K: phosphatidylinositol 3-kinase. AKT: serine threonine kinase. BIT: baseline incubation temperature, 40 °C. HT: high temperature, 44 °C. PI3K-IN-1: PI3K/AKT inhibitor. YS-49: PI3K/AKT agonist. Values of phosphorylated protein expression levels of target genes were calculated as the relative quantities (RQ) of phosphorylated protein band intensities of target genes to their corresponding protein band intensities. Different symbols ($, *, #) indicate significant differences (*p* < 0.05) between BIT and HT for the same JO type with no Zn addition. Different letters (A, B, C) indicate significant differences (*p* < 0.05) among the three JO types under BIT. Different letters (a~f) indicate significant differences (*p* < 0.05) among treatments under HT. Data are means ± SE (n = 4–6). N = 4–6, because there are one or two replicate outliers for some treatments, and we adopted the commonly-used interquartile range (IQR) method to eliminate outliers within each group.

Figure 11A,B showed that, under BIT, the JO type impacted (*p* < 0.001) the phosphorylated protein expression levels of both PI3K and AKT in JOs. The IE JOs decreased (*p* < 0.001) them compared with the NE JOs, while the AE JOs increased (*p* < 0.001) them. HT reduced (*p* < 0.001) them in the three JO types with no Zn addition compared with BIT. The JO type, Zn source and their interaction had effects (*p* < 0.001) on them under HT. In NE and AE JOs, supplemental Zn, especially Zn-Prot M, increased (*p* < 0.05) them compared with no Zn addition, but the increasing effect was further expanded (*p* < 0.05) in AE JOs compared to NE JOs. However, in IE JOs, Zn supplementation had no effect (*p* > 0.05) on them.

## 4. Discussion

The results from the current study indicate that inhibiting or activating the PI3K/AKT signaling pathway remarkably inhibited or enhanced the mitigating effect of Zn, especially Zn-Prot M, on HS-induced damage to the integrity and barrier function of JOs. The inhibition or enhancement appeared to be associated with the decreased or increased promotion of cell proliferation, supporting our hypothesis. To our knowledge, these findings have not been previously reported in poultry or other animals, thus providing new targets and scientific support for the effective mitigation of HS-induced damage to the intestinal integrity and barrier function through dietary Zn-Prot M supplementation and other feed strategies in broiler production.

The effective concentrations of inhibitors and agonists differ between cell types [37]. Both PI3K-IN-1 and YS-49 are the specific inhibitor and agonist of the PI3K/AKT signaling pathway, respectively. Studies on gastric cancer cells and lung cancer cells have shown that supplementation of 25 μmol/L of PI3K-IN-1 in the medium reduced the phosphorylation of PI3K and AKT [31,32]. Addition of 10 μmol/L of YS-49 increased the phosphorylation of PI3K and AKT in BPJECs [30]. In the current study, 16 μmol/L was chosen to be the optimal supplemental level of PI3K-IN-1 to ensure the effective inhibition of both PI3K and AKT phosphorylation with the minimum negative effect on the integrity, barrier function and budding percentage of JOs. Meanwhile, 9 μmol/L was chosen to be the optimal supplemental level of YS-49 to ensure the effective promotion of both PI3K and AKT phosphorylation in JOs.

We utilized the above optimal supplemental levels of PI3K-IN-1 and YS-49 to further investigate the phosphorylation regulation of the PI3K/AKT signaling pathway and its involvement in the Zn alleviation of HS-induced damage to broiler JOs via promotion of cell proliferation. The same results as above were obtained, further confirming the effectiveness of the PI3K/AKT inhibition or activation induced by PI3K-IN-1 or YS-49 in JOs. Previous studies have indicated that in mouse intestinal epithelial cells, the addition of a PI3K/AKT inhibitor effectively blocked the promoting effect of low-dose boron on cell proliferation and damaged the barrier function [38]. In a dextran sulfate sodium (DSS)-induced mouse colitis model, excessive activation of the pathway by adding PI3K/AKT agonist led to the abnormal proliferation of intestinal epithelial cells, while the inhibition of the PI3K/AKT signaling pathway by astragaloside eliminated the abnormal proliferation and repaired the intestinal barrier dysfunction [39]. Adding an inhibitor or agonist of the PI3K/AKT signaling pathway decreased or enhanced the protective effect of Zn on HS-induced damage to BPJECs by the inhibited or promoted cell proliferation [30]. In the present study, with no Zn supplementation, HS significantly impaired the integrity, barrier function and budding percentage of JOs and reduced cell proliferation, as well as the phosphorylation of PI3K and AKT regardless of the JO type, which is consistent with our previous work [13]. Under HT, compared to NE, IE markedly aggravated the damage to the barrier function of JOs, which might be related to decreased cell proliferation as a result of inhibitor addition. However, AE significantly alleviated the impairment to barrier function of JOs, which might be associated with the increased cell proliferation after adding the agonist. More importantly, the inhibition of PI3K/AKT strongly weakened the alleviating effect of Zn supplementation on HS-induced damage to JOs, while activation of this pathway further strengthened the HS-resisting effect of Zn, especially Zn-Prot M via the inhibition or promotion of cell proliferation. These results are in agreement with the other reports above [30,38,39]. Zn-Prot M outperformed ZnSO_4_ on all the aforementioned aspects, probably owing to its higher Zn absorption and higher bioavailability in broilers [40,41,42,43,44]. The above results indicate that Zn, especially Zn-Prot M, can upregulate the expression of the PI3K/AKT signaling pathway to promote cell proliferation and alleviate HS-induced damage to the integrity and barrier function of JOs or BPJECs.

## 5. Conclusions

To our knowledge, this study has, for the first time, confirmed the involvement of the PI3K/AKT pathway in Zn (particularly Zn-Prot M) alleviation of HS-induced damage to the integrity and barrier function of broiler JOs via promotion of cell proliferation. However, the upstream link between Zn and the PI3K/AKT pathway is lacking. Our recent studies suggest that the GPR39-mediated PI3K/AKT pathway would possibly participate in Zn (particularly Zn-Prot M) alleviation of HS-induced damage to the broiler jejunum and JOs [13,34]. The GPR39 is a G-coupled Zn-binding receptor that senses Zn changes in the extracellular environment and that functions in mediating signal transduction [34]. Therefore, further investigation is needed to confirm the role of the above pathway in this Zn alleviation process via GPR39 silencing and overexpression.

## Figures and Tables

**Table 1:** Analyzed Zn concentrations in the media of all treatment groups (Exp. 2).

Group	Supplemental Zn (μmol/L)	Measured Zn (μmol/L) ^1^
Basal Zn (no Zn addition)	0	9.4
ZnSO_4_	50	60.8
Zn-Prot M ^2^	50	60.9

^1^ Data are the analyzed values based on triplicate measurements. ^2^ Zn-Prot M: Zn proteinate with moderate chelation strength (Q_f_ = 51.6).

**Table 2:** List of primary antibodies.

Protein	Dilution Ratio	No.	Company	City/Country
PI3K ^1^	1:1000	A19742	ABclonal	Beijing/China
AKT ^2^	1:1000	AF6261	Affinity	Liyang/China
p-PI3K ^3^	1:1000	bs-6417R	Bioss	Beijing/China
p-AKT ^4^	1:1000	bs-0876R	Bioss	China

^1^ PI3K: phosphatidylinositol 3-kinase. ^2^ AKT: serine threonine kinase. ^3^ p-PI3K: phosphorylated phosphatidylinositol 3-kinase. ^4^ p-AKT: phosphorylated serine threonine kinase.

## Data Availability

The data presented in this study are available in the article.
